# Green Tea and One of Its Constituents, Epigallocatechine-3-gallate, Are Potent Inhibitors of Human 11β-hydroxysteroid Dehydrogenase Type 1

**DOI:** 10.1371/journal.pone.0084468

**Published:** 2014-01-03

**Authors:** Jan Hintzpeter, Claudia Stapelfeld, Christine Loerz, Hans-Joerg Martin, Edmund Maser

**Affiliations:** Institute of Toxicology and Pharmacology for Natural Scientists, University Medical School Schleswig-Holstein, Campus Kiel, Germany; Fudan University, China

## Abstract

The microsomal enzyme 11β-hydroxysteroid deydrogenase type 1 (11β-HSD1) catalyzes the interconversion of glucocorticoid receptor-inert cortisone to receptor- active cortisol, thereby acting as an intracellular switch for regulating the access of glucocorticoid hormones to the glucocorticoid receptor. There is strong evidence for an important aetiological role of 11β-HSD1 in various metabolic disorders including insulin resistance, diabetes type 2, hypertension, dyslipidemia and obesity. Hence, modulation of 11β-HSD1 activity with selective inhibitors is being pursued as a new therapeutic approach for the treatment of the metabolic syndrome. Since tea has been associated with health benefits for thousands of years, we sought to elucidate the active principle in tea with regard to diabetes type 2 prevention. Several teas and tea specific polyphenolic compounds were tested for their possible inhibition of cortisone reduction with human liver microsomes and purified human 11β-HSD1. Indeed we found that tea extracts inhibited 11β-HSD1 mediated cortisone reduction, where green tea exhibited the highest inhibitory potency with an IC50 value of 3.749 mg dried tea leaves per ml. Consequently, major polyphenolic compounds from green tea, in particular catechins were tested with the same systems. (−)-Epigallocatechin gallate (EGCG) revealed the highest inhibition of 11β-HSD1 activity (reduction: IC50 = 57.99 µM; oxidation: IC50 = 131.2 µM). Detailed kinetic studies indicate a direct competition mode of EGCG, with substrate and/or cofactor binding. Inhibition constants of EGCG on cortisone reduction were Ki = 22.68 µM for microsomes and Ki = 18.74 µM for purified 11β-HSD1. *In silicio* docking studies support the view that EGCG binds directly to the active site of 11β-HSD1 by forming a hydrogen bond with Lys187 of the catalytic triade. Our study is the first to provide evidence that the health benefits of green tea and its polyphenolic compounds may be attributed to an inhibition of the cortisol producing enzyme 11β-HSD1.

## Introduction

Tea *(Camellia sinensis)* is the second most widely consumed beverage in the world after water [1] and has been cultivated for thousands of years due to its medicinal benefits and general health promotion purposes.

The tea plant is naturally occurring in South China, but is nowadays cultivated in many other regions of the major tea producing countries in the world, like India, Japan, Sri Lanka, Indonesia and Kenia. In general, tea can be divided into three types (percentage of worlds tea production): non-fermented green tea (20%), semi-fermented tea (e.g. oolong tea and white tea) (2%) and fermented black tea (78%) [2]. Additionally, there are more than 300 different kinds of tea that differ regarding the manufacturing process.

The most popular form of tea consumed in the world is black tea, whereas green tea is mainly consumed in China and Japan. Recently, plenty of commercial beverages came to market that contain tea extracts or catechins from tea. Nowadays, tea or beverages containing tea extracts, if consumed daily, belong to a life-style that might support healthiness and long life, which is underpined by several laboratory, epidemiological and human intervention studies [3]–[7].

In particular, consumption of green tea has been associated with a reduction of the risk of cardiovascular disease, some forms of cancer, as well as with the promotion of oral health and other physiological functions such as antibacterial and antiviral activity, neuroprotective properties, anti-hypertensive effects, body weight control and diabetes type 2 prevention [8], [9].

The latter diseases are risk factors of the metabolic syndrome (obesity, insulin resistance, hypertension, diabetes type 2, dyslipidemia) against which the therapeutical potential of tea has been shown in humans and model organisms in numerous studies [10]–[14].

In most cases the beneficial effects have been attributed to the polyphenolic compounds, especially catechins, although a large number of potentially bioactive chemicals are present in tea as well [15]. (−)-Epigallocatechin-3-gallate (EGCG) is the major component among the tea catechins and is believed to have a considerable therapeutical potential [16].

Unlike semi-fermented and fermented teas (black and white teas) unfermented green tea contains more catechins [17]. A typical green tea infusion of 250 ml hot water with 2.5 g tea leaves approximately contains 620–880 mg of water-extractable solid compounds. About 30–42% of the dry weight of green tea consists of phenolic compounds [18], [19], from which EGCG is the most abundant one (up to 50–80% of the total catechins [18]).

Other catechins are present in smaller amounts: (−)-epigallocatechin (EGC)>(−)-epicatechin gallate (ECG)>(−)-epicatechin (EC)> gallocatechin gallate (GCG)> gallocatechin (GC)> catechin gallate (CG)> catechin (C)> epigallocatechin digallate > epicatechin digallate [18].

Many other components have been identified in tea that might have an effect on human health: theaflavins, thearubigins, theasinesins, gallic acid, quinic acid, theogallin, coumaryl-quinic acid, caffeine, theobromine and theophylline, L-theanine (unique to tea), kaempferol, myricetin and quercetin [2], [17].

It has been estimated that up to one-third of patients with diabetes type 2, hypertension and dyslipidaemia consume some form of complementary and alternative medicine, involving the use of herbs and other dietary supplements, like plant infusions, as alternatives to mainstream Western medical treatment [20]. For this reason, our lab is in search of herbs that are traditionally used for the treatment of symptoms of the metabolic syndrome in the medicine of several indigenous populations as well as in traditional Chinese medicine. In comparison with high-throughput screening of large chemical libraries to discover new drugs, medical treatments of chronic diseases by traditional Chinese medicine and indigenous populations feature clear benefits based on their thousand-year-old experience in dealing with some of the above mentioned chronic diseases [21].

**Figure 1:**
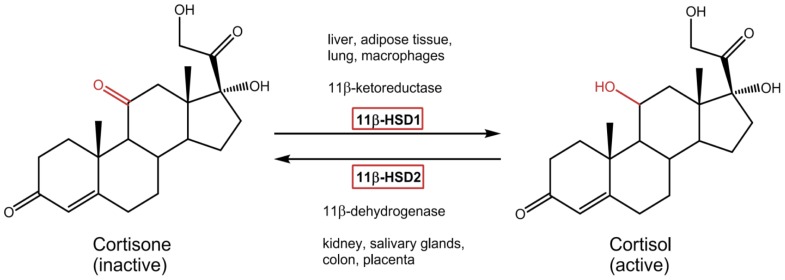
Physiological role of the two isoenzymes 11β-HSD type 1 and 2. Predominant reaction direction of the NADP(H)-dependent enzyme 11β-HSD1 by catalyzing the conversion of inactive cortisone to receptor-active cortisol. The reverse reaction is mediated by the unidirectional NAD-dependent 11β-HSD type 2.

In humans high levels of intracellular cortisol can increase glucose output in the liver, induce fat accumulation and weaken insulin sensitivity in adipose tissues, resulting in an increased risk for the metabolic syndrome [22]. Cortisol-levels are regulated by two known isoforms of 11β-hydroxysteroid dehydrogenase (see Figure 1): a microsomal NADPH-dependent 11β-hydroxysteroid dehydrogenase type 1 (11β-HSD1) primarily acting as a reductase in liver, fat tissues, lung and macrophages and a NAD dependent 11β-hydroxysteroid dehydrogenase type 2 (11β-HSD2) that is expressed in kidney, salivary glands, colon and placenta. 11β-HSD2 is a unidirectional oxidase that deactivates glucocorticoid-receptor active cortisol to cortisone [23]–[26].

Multiple evidence argues for an important aetiological role of 11β-HSD1 in various metabolic disorders including insulin resistance or rather diabetes type 2, hypertension, dyslipidaemia and obesity [22], [27]–[31]. 11β-HSD1-deficient mice show enhanced glucose tolerance, improved hepatic insulin resistance, attenuated gluconeogenesis, and an improved lipid and lipoprotein profile [32]–[34]. Moreover, overexpression of 11β-HSD1 selectively in adipose tissue causes visceral obesity, insulin resistance, type 2 diabetes, dyslipidemia, and hypertension in mice [22].

Collectively, these findings emphasize the potential benefits of a specific inhibitor of human 11β-HSD1 in the treatment of obesity and type 2 diabetes. In the present investigation, we tested several teas and some major tea-specific polyphenolic compounds, catechins, for their capability to inhibit the cortisol producing enzyme 11β-HSD1. Inhibition of 11β-HSD1 would shed light on – at least some of – the mechanisms by which green tea performs its health effect.

## Materials and Methods

### Ethics Statement

Work on pluripotent carbonyl reductases/hydroxysteroid dehydrogenases, including 11β-HSD1, was approved by the ethics commission of the Medical Faculty, University of Kiel. Human liver tissue was obtained with written informed consent and approval by ethics commission of the Medical Faculty, University of Kiel. All experimental studies were performed in accordance with German legislation.

### Chemicals

Cortisol, cortisone and EGCG were purchased from Sigma-Aldrich (St. Louis, MO). The following chemicals were procured from Carl Roth GmbH + Co. KG (Karlsruhe, Germany): Ethylacetate, NADP, NADPH, non-fat dry milkpowder, monosodium phosphate and potassium chloride, HPLC column LiChrospher® 100 RP-18, methanol, sodium chloride and monopotassium phosphate were obtained from Merck (Darmstadt, Germany). (−)-Epicatechin gallate, (−)-catechin and (−)-gallocatechin were purchased from Biomol GmbH (Hamburg, Germany), Cayman Chemical (Ann Arbor, MI) and LKT Laboratories Inc. (St. Paul, MN), respectively. The different types of commercially available teas consisting of green, oolong and black teas were bought from supermarkets in Kiel, Germany. The anti-HSD11B1 antibody (ab83522) was obtained from Abcam (Cambridge, UK).

### Preparation of tea extracts

Dried tea leaves were reduced to small pieces and 1 g of the crushed material was suspended in 10 mL water without stirring for 90 min at room temperature. Insoluble material was removed by centrifugation (13,000 RPM in a microcentrifuge) and the resulting supernatant was used in enzyme inhibition experiments.

### Preparation of 11β-HSD1-containing microsomes and purification of human 11β-HSD1

Human liver microsomes and purified human 11β-HSD1 were prepared as described previously [35], [24].

### Enzyme assay

In terms of 11β-HSD1 reductase activity, inhibition analysis was performed as follows: 50 µl of tea extract were added to a mixture containing 400 µM cortisone, 0.8 mM NADPH solution in 100 mM phosphate buffer pH 7.4. The reaction was started by adding 5 µl of human liver microsomes (218 µg protein) and the mixture was incubated for 3 h at 37°C. The total assay volume was 250 µl.

The reaction was stopped by adding 250 µl ice-cold ethylacetate and vortexing, followed by a 30 sec centrifugation at 13,000 RPM in a microcentrifuge. The organic phase was separated and the aqueous phase extracted two more times with 250 µl ethylacetate. The ethylacetate phases were combined and the solvent was removed in a SpeedVac (ThermoSavant). The resulting residue was dissolved in 150 µl methanol-water (58∶42, v/v) and used for metabolite quantification by HPLC.

To analyze inhibition of 11β-HSD1 dehydrogenase activity, the oxidation of cortisol to cortisone was measured essentially as described above, except for the substitution of NADPH with 0.8 mM NADP, and the use of 400 µM cortisol instead of cortisone. Here, the incubation time was 60 min at 37°C in a 100 mM phosphate buffer pH 9.0.

### Metabolite quantification by HPLC

After enzymatic conversion the glucocorticoids were resolved as published previously [36]. Product yield was quantified by peak integration of the cortisone and cortisol peaks, respectively.

### Determination of the mode of 11β-HSD1 inhibition by EGCG and of its inhibition constant Ki

The type of inhibition by EGCG (dissolved in buffer solution) was determined with four inhibitor (0, 20, 50, 75, 100 µM EGCG) and substrate (50, 100, 200, 400 µM cortisone) concentrations. The data obtained were plotted as substrate-velocity curves and analyzed by nonlinear-regression. The type of inhibition was tested with both human liver microsomes (218 µg) and purified 11β-HSD1 (16.4 µg).

### Western Blot analysis after EGCG incubation with microsomal and purified 11β-HSD1

To exclude EGCG-induced 11β-HSD1 aggregate formation, purified 11β-HSD1 (2.14 µg) was preincubated with 25–100 µM EGCG (3 h at 37°C) and then prepared for sodium dodecyl sulfate polyacrylamide gel electrophoresis (SDS-PAGE) according to the manufacturer's protocol. As a control, the same volume of untreated purified protein was loaded onto the parallel lane.

The resolved proteins were transferred to nitrocellulose membranes. After blocking the membrane for 1 h at RT with 5% non-fat dry milkpowder in PBS-buffer, the blocking solution was replaced by the primary antibody at a 1∶2,000 dilution and the membrane was incubated at 4°C for about 16 h. Primary and secondary antibodies were diluted in PBS containing 2.5% non-fat dry milkpowder. The membrane was washed three times and then the corresponding secondary antibody was added at a dilution of 1∶10,000. After a 1.5 h incubation at RT the membrane was washed three times and the specific Western blot signals of 11β-HSD1 were detected by the ECL Western Blotting System according to the manufacturer's instructions.

### Quantification of hydrogen peroxide by xylenol orange

According to Halliwell [37] and Lay et al. [38], EGCG induces hydrogen peroxide production in various buffer systems. For this reason, we determined the hydrogen peroxide concentration produced by EGCG (concentration 200 µM) after 3 h incubation at 37°C either in the buffer used or in our complete assay systems [39]. Further, to exclude any inhibitory effects of hydrogen peroxide on cortisone reduction, we tested the influence of hydrogen peroxide on cortisol production by HPLC (as described above) with both human liver microsomes and purified 11β-HSD1, respectively.

## Results

### Concentration-dependent inhibition of cortisol production by three types of tea in human liver microsomes

**Figure 2:**
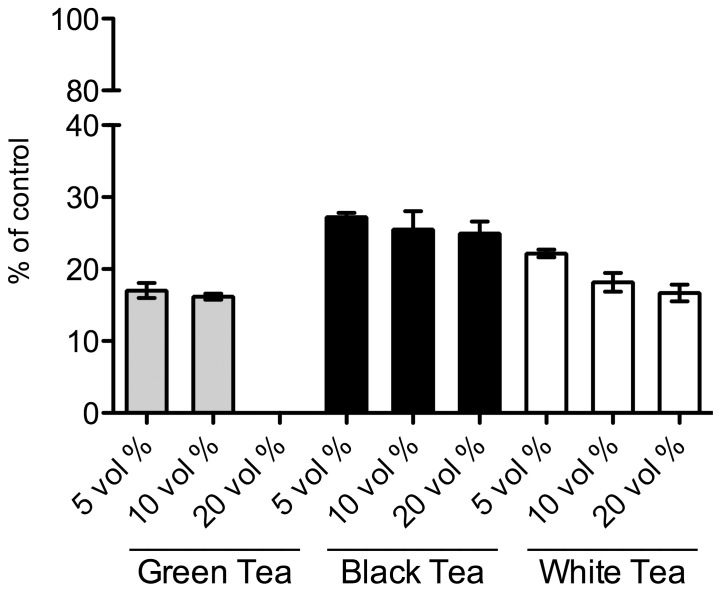
Inhibition of cortisone reduction by three different types of tea (Green, Black and White Tea) in human liver microsomes. Product yields were quantified by integration of cortisol peaks. The product yields of the controls without any type of tea were normalized to 100% enzyme activity and the residual activity expressed as percent of the control. Bars represent the mean ±SD of at least three repeat experiments.

Inhibition of 11β-HSD1 activity was assessed by determining cortisol yields after 3 h incubation at 37°C with equal volumes of green, black and white tea infusions (100 mg tea per ml; prepared as described above). Quantification was performed by integration of the well-resolved product peaks in the above discribed HPLC assay. Figure 2 shows the inhibitory effects of three commercially available teas on human microsomal 11β-HSD1 reductase activity. Conversion of cortisone to cortisol was inhibited by all three types of tea in a concentration-dependent manner, with green tea exhibiting the highest inhibition.

### Concentration-dependent inhibition of cortisol production by green tea in human liver microsomes

**Figure 3:**
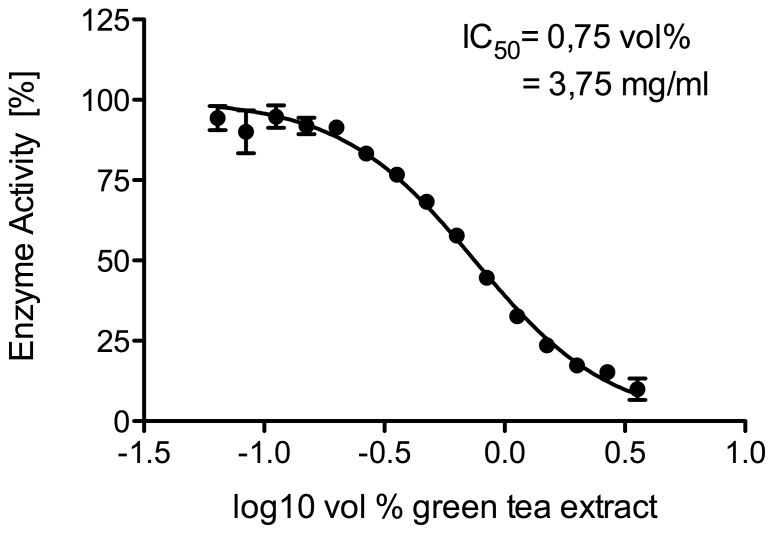
Dose-dependent inhibition of cortisone reduction by green tea in human liver microsomes. Results are presented as means ±SD (n = 4).

As shown in Figure 2, green tea extract displayed the highest inhibitory potency of the three types of tea tested. With human liver microsomes and different amounts of green tea extract a dose-response inhibition curve of microsomal cortisone reduction was observed. An IC50-value of 3.75 mg crushed dried tea leaves per ml (this corresponds to a value of 0.75 vol% of green tea extract in the reaction mixture) was determined (Fig. 3).

### Inhibition of cortisol production by several constituents of green tea in human liver microsomes

**Figure 4:**
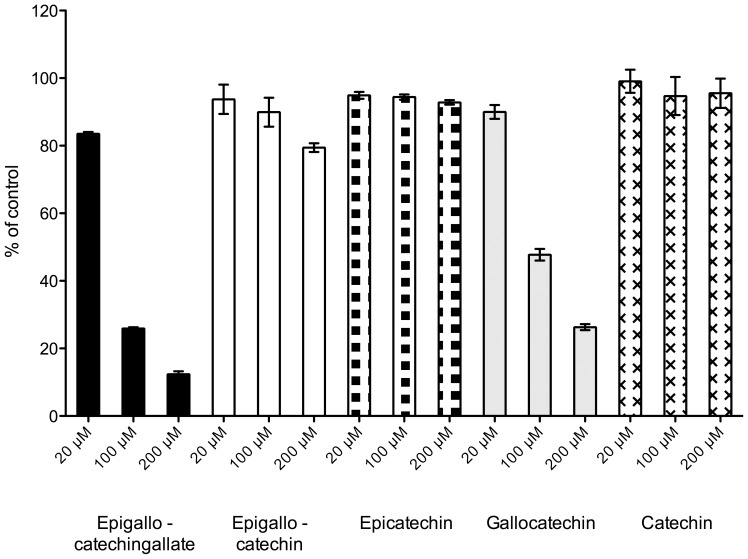
Inhibitory effects of five tea catechins on cortisone reduction in human liver microsomes. Bars represent the mean ±SD of at least three repeat experiments.

**Figure 5:**
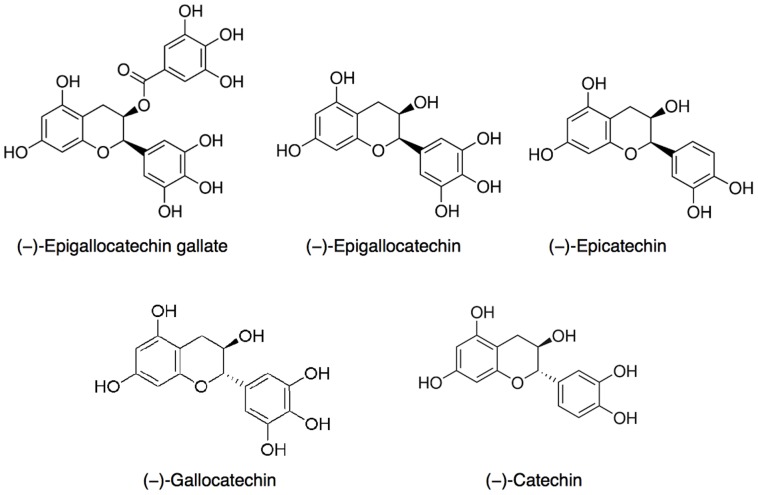
Chemical structures of five green tea catechins used in this study. EGCG, (−)-epigallocatechin-3-gallate; EGC, (−)-epigallocatechin; EC, (−)-epicatechin; GC, (−)-gallocatechin; C, (−)-catechin.

**Figure 6:**
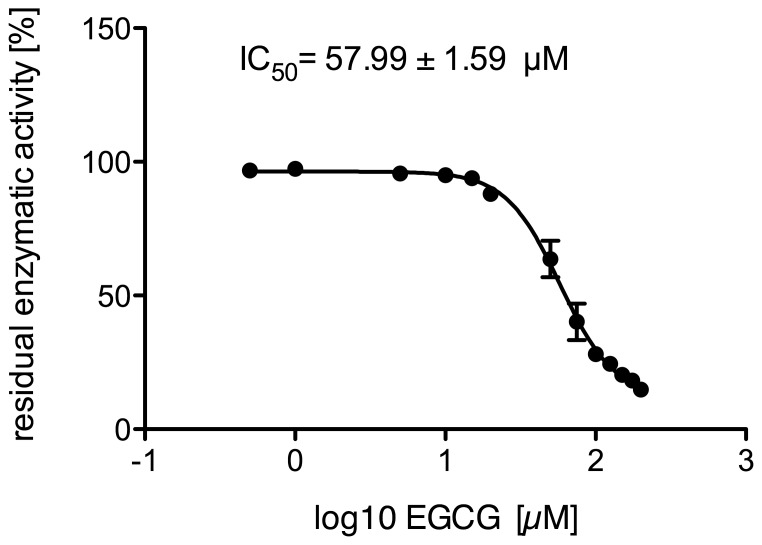
Dose-dependent inhibition of cortisone reduction by EGCG in human liver microsomes. Results are presented as means ±SD (n = 4).

Inhibition of cortisone reduction in human liver microsomes was assessed with several green tea constituents. Figure 4 shows the residual enzymatic activity after incubation with five catechins found in green tea (see Fig. 5) measured at three concentrations. Highest inhibition was achieved with EGCG, followed by GC (gallocatechin). The half maximal inhibitory concentration (IC50) determined for EGCG on 11β-HSD1 activity in microsomes was 57.99 µM (Figure 6). It should be noted that an aqueous solution of EGCG, when stored at −20°C for one week, resulted in an IC50-value about half as high (26.88 µM) as that determined with freshly prepared EGCG solutions.

**Figure 7:**
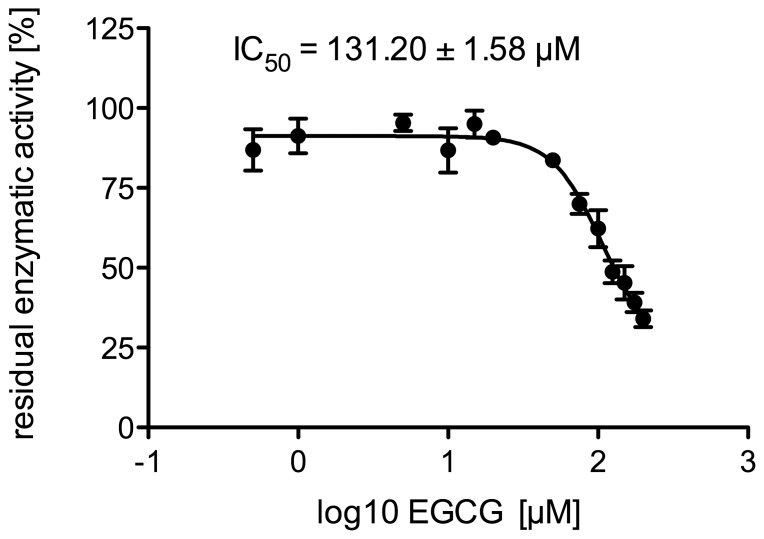
Dose-dependent inhibition of cortisol oxidation by EGCG in human liver microsomes. Results are presented as means ±SD (n = 4).

To determine possible differences in the potential of EGCG to inhibit reductase and dehydrogenase activity of 11β-HSD1, the influence of EGCG on the oxidation of cortisol to cortisone was monitored. Figure 7 shows the EGCG concentration-dependent decrease of 11β-HSD1 cortisol dehydrogenase activity in microsomes. From these data an IC50-value of 131.20 µM was calculated.

### Determination of the type of inhibition by EGCG and of its dissociation constant Ki with human liver microsomes and purified 11β-HSD1

**Figure 8:**
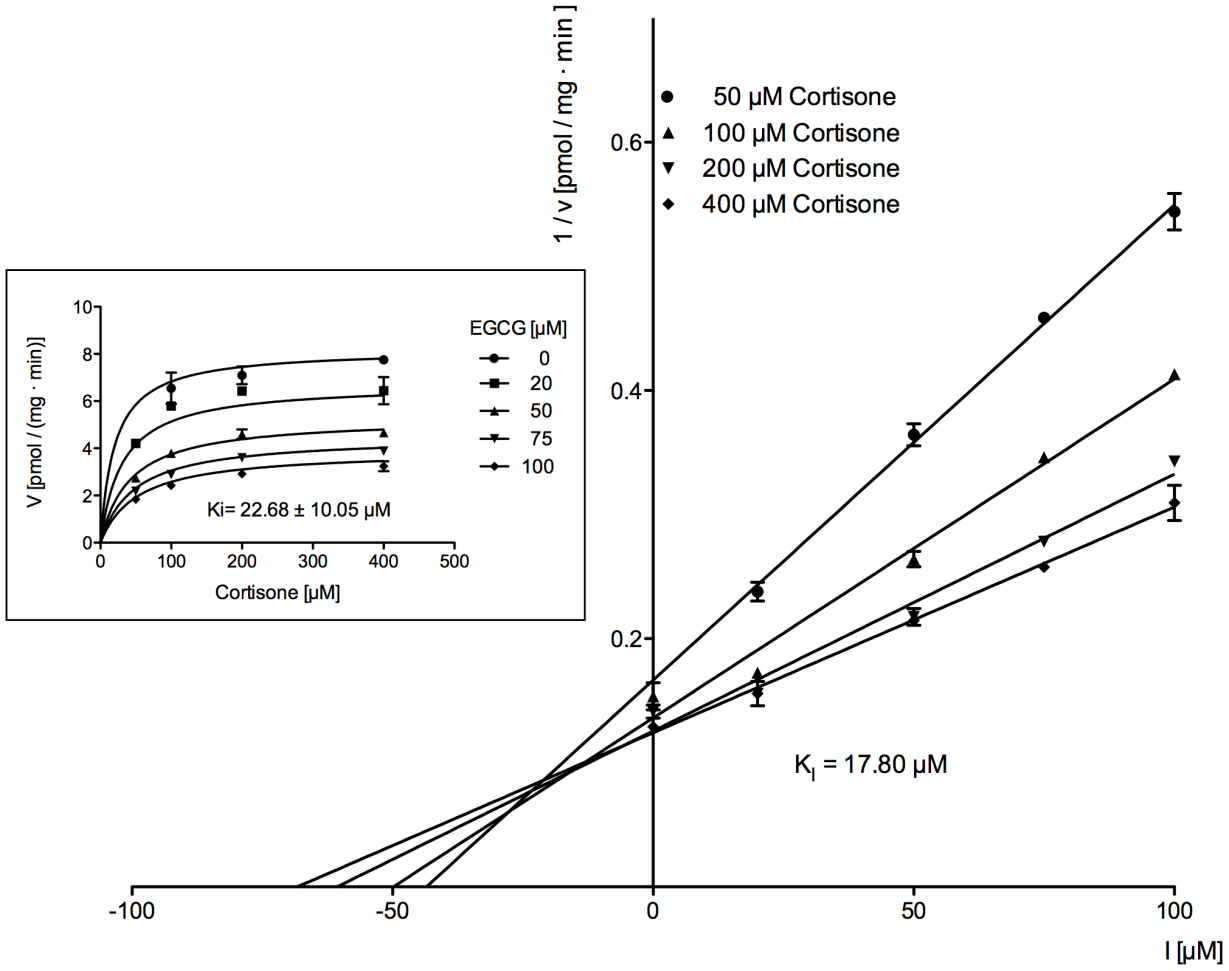
Dixon plot to characterize the inhibition type and to determine the Ki of EGCG on cortisone reduction in human liver microsomes. The inset shows direct plotting of the same data.

**Figure 9:**
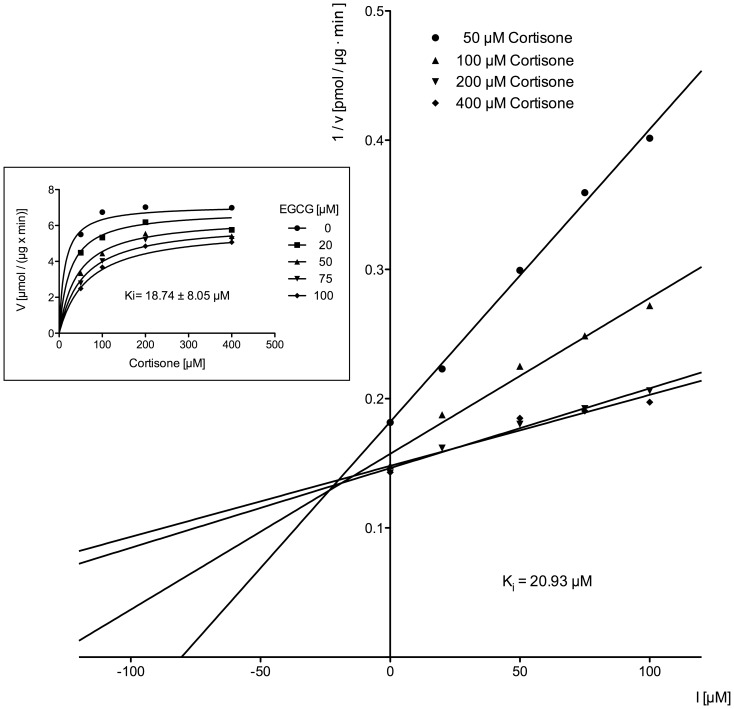
Dixon plot to characterize the inhibition type and to determine the Ki of EGCG on cortisone reduction by purified 11β-HSD1. The inset shows direct plotting of the same data.

To determine the mechanism of 11β-HSD1 inhibition by EGCG various inhibitor and substrate concentrations were used. Non-linear regression analysis yielded Ki -values of Ki = 22.68±10.10 µM and Ki = 18.74±8.00 µM for microsomes and the purified enzyme, respectively (Insets Figs. 8,9 and Table 1). The data were best fitted using a model of mixed inhibition. In line with this calculation were the results of the Dixon-plots (Figures 8,9). The regression lines converge above the abscissa indicating a competetive or mixed mode of inhibition and Ki -values similar to those obtained by non-linear regression (Table 1).

**Table 1:** Inhibition constants of EGCG on cortisone reduction with human liver microsomes and purified human 11β-HSD1.

	human liver microsomes	purified human 11β-HSD1
Ki (non-linear regression)	22.68 µM	18.74 µM
Ki (estimated by Dixon-plot)	17.80 µM	20.93 µM

**Figure 10:**
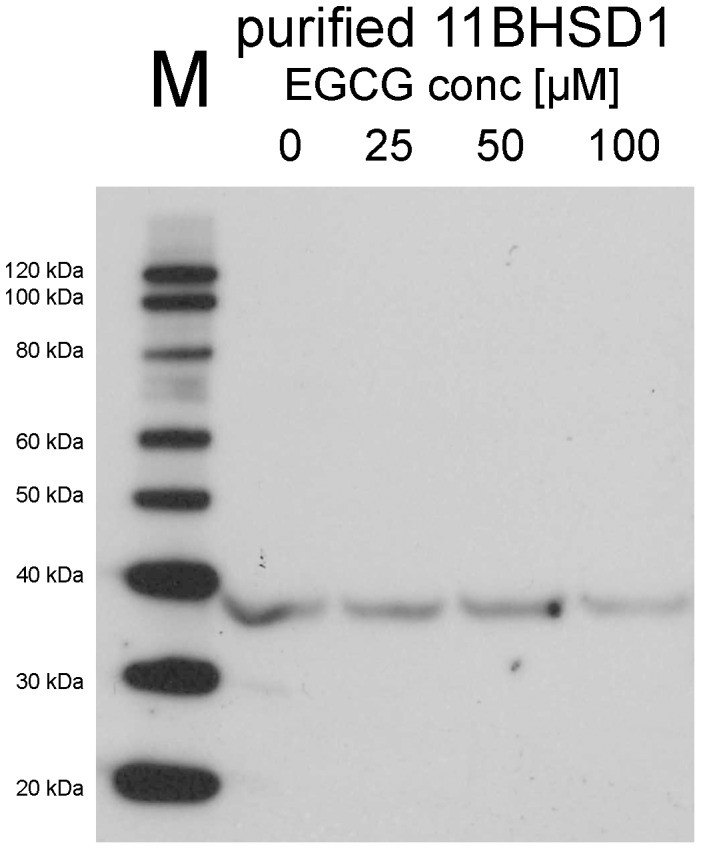
Western Blot analysis of EGCG treated and EGCG untreated purified human liver 11β-HSD1.

**Figure 11:**
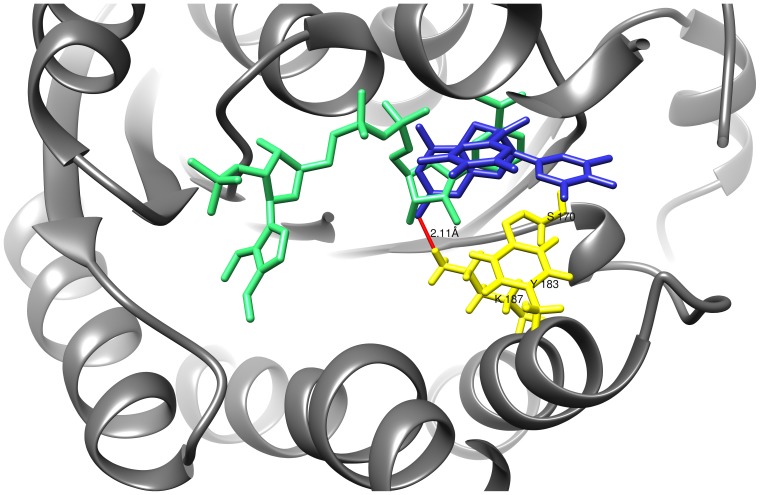
Binding model of EGCG docked to the active site of human 11β-HSD1. Shown is a binary complex of 11β-HSD1 (PDB: 2RBE) with EGCG (blue) and NADPH (green). K187 belongs to the catalytic triade (yellow) and forms a predicted hydrogen bond with EGCG (distance: 2.1 Å).

Data are derived from non-linear regression curves and Dixon-plots (Figs. 8–11).

### Western Blot analysis after EGCG incubation with purified 11β-HSD1

As shown in Figure 10, EGCG preincubation (3 h at 37°C) with purified 11β-HSD1 did not induce protein aggregate formation. No differences in the treated (lane 2–4) and untreated samples (lane 1) of purified 11β-HSD1 were observed, even at EGCG concentrations up to 100 µM.

### Quantification of EGCG generated hydrogen peroxide by xylenol orange

When 200 µM EGCG was incubated for 3 h at 37°C in buffer only (same buffer as used in our assays above), the formation of 220 µM hydrogen peroxide was determined by the xylenol orange assay. However, when human liver microsomes, NADPH and cortisone was added (in the same concentrations as in the enzyme assays), only 12 µM hydrogen peroxide could be detected. Further, we tested the influence of up to 300 µM hydrogen peroxide on cortisol production with both human liver microsomes and purified human 11β-HSD1, respectively. No 11β-HSD1 inhibition by hydrogen peroxide could be detected (data not shown).

## Discusssion

Tea infusions have not only been a popular beverage for thousands of years but also have been traditionally used for the treatment of the metabolic syndrome and other chronic diseases. The metabolic syndrome is a complex metabolic disorder that may lead to diabetes type 2 and is one of the fastest growing diseases within the western civilisation. The role of glucocorticoid hormones in maintaining the blood glucose level by mobilization of energy sources is essential for homeostasis: Cortisol deficiency results in low blood glucose levels, dizziness, generalized weakness and stress sensitivity while cortisol overproduction, hypercortisolism, leads to high blood pressure, increased abdominal fat deposition, insulin resistance etc. Due to its pivotal role in cortisol production, inhibition of 11β-HSD1 maybe of therapeutic value in the treatment of the metabolic syndrome and diabetes.

For these reasons our laboratory has investigated the inhibitory effects of tea, in particular green tea and some of its constitutents, on microsomal and purified 11β-HSD1 mediated cortisone reductase (and cortisol dehydrogenase) activity. The present study demonstrates that tea and green tea, respectively, and some of their polyphenolic constituents, EGCG and GC, have the capability to inhibit human 11β-HSD1.

Initially, we could show that non-fermented green tea, semi-fermented tea (e.g. oolong tea and white tea) and fermented black tea have different potencies to inhibit human 11β-HSD1 mediated cortisone reduction. Non-fermented green tea showed the highest inhibitory potential (IC50 of 3.75 mg crushed dried tea leaves per ml, which corresponds to a value of 0.75 vol% of green tea extract), whereas a weaker inhibition potency was observed by semi-fermented white and fermented black tea.

A reason for higher 11β-HSD1 inhibition by green tea compared to other teas might be a higher concentration of phenolic compounds in the former. During the fermentation process phenolic compounds, like catechins, are enzymatically oxidized. To protect the polyphenolic compounds from enzymatic oxidation, tea leaves that are destined to become green tea are withered by air drying prior to heat inactivation of polyphenol oxidases.

Consequently, we tested several polyphenolic compounds known from green tea, in particular catechins, for their inhibition of 11β-HSD1 cortisone reduction. Interestingly, we observed big differences in the inhibition potency among the structurally related catechins (see Figure 5). For example, (−)-epigallocatechin, (−)-epicatechin and (−)-catechin showed almost no or very little inhibition ((−)-epigallocatechin ca. 25% inhibition at 200 µM). However, when (−)-epigallocatechin gallate (ca. 75% inhibition at 100 µM) and (−)-gallocatechin (ca. 53% inhibition at 100 µM) were used as inhibitors, a much larger effect could be detected. A possible explanation might be that all other catechins tested in this study are non-gallic acid esterified compounds (Fig. 5). (−)-Epigallocatechin and (−)-gallocatechin are diastereoisomers (Fig. 5) from which (−)-gallocatechin showed a greater inhibition (Fig. 4). The gallic acid esterified form of (−)-epigallocatechin, EGCG, is the most potent substance tested so far, which may lead to the assumption that the gallic acid ester form of (−)-gallocatechin, (−)-gallocatechin gallate may exhibit an even greater inhibitory potential than EGCG. Nevertheless, EGCG showed the highest inhibition in this study and was an obvious choice for further investigation.

From cell culture experiments it is known that polyphenols like EGCG and EGC trigger a rapid hydrogen peroxide generation in various buffer systems. Concentrations up to 220 µM of hydrogen peroxide generated by 200 µM EGCG (incubated for 3 h at 37°C) in the buffer systems used could be detected. In contrast, addition of human liver microsomes, NADPH and cortisone to the reaction mixture resulted in about 12 µM hydrogen peroxide. A possible explanation for this hydrogen peroxide preventing effect might be the presence of peroxisomes in the microsomal fraction which contain several peroxidases known to catalyze the reduction of hydrogen peroxide to water. Further, due to the possibility that hydrogen peroxide generated by catechins could be responsible for the 11β-HSD1 inhibition observed in our study, we tested the inhibitory effects of up to 300 µM hydrogen peroxide on cortisone reduction. However, no inhibition of cortisol formation by both human liver microsomes and purified human 11β-HSD1, respectively, could be detected.

Next, we determined the IC50-values of EGCG to inhibit the conversion of cortisone to cortisol and *vice versa* by human liver microsomes. The results indicate that the dehydrogenase activity seems to be less affected by EGCG than the reducing activity (reduction: IC50 = 57.99 µM; oxidation: IC50 = 131.20 µM; see Figs. 6, 7).

As mentioned before, 11β-HSD1 reductase and dehydrogenase activities are varying from tissue to tissue [25]. Moreover, recent reports indicate the capability of cortisol oxidation by 11β-HSD1 in subcutaneous adipose tissues [40]. Hence, a shift in the equilibrium towards cortisone may occur *in vivo*. In the past, selectivity of inhibitors for 11β-HSD1 over 11β-HSD2 has received most attention. In addition, effectiveness of inhibitors should also consider a selectivity for cortisone reduction by 11β-HSD1 over its cortisol to cortisone dehydrogenase reaction. This assumption may be underpined by the fact that many selective 11β-HSD1 inhibitors failed in clinical trials [41].

A recently published paper about the inhibition of cortisol production by EGCG in rat liver microsomes proposes a redox shift in the lumen of the endoplasmic reticulum (ER) being responsible for the EGCG dependent effect [42]. However, in our present study we used purified 11β-HSD1 from human liver for EGCG inhibition studies. Detailed kinetic analysis yielded a mixed mode of EGCG on 11β-HSD1 inhibition with respect to cortisone. Moreover, we were able to determine a Ki for EGCG of 18.74 µM using non-linear regression analysis, which is similar to the Ki determined by using human liver microsomes (Ki = 17.8 µM). As a consequence, our results indicate a direct effect of EGCG to both purified and microsomal 11β-HSD1 rather than a EGCG-induced shift of NADPH to NADP in the ER lumen. To strengthen our findings, EGCG was docked into the X-ray crystal structure of human 11β-HSD1 (PDB: 2RBE) using SwissDock [43]. The result of EGCG positioned into the active site of 11β-HSD1 (Figure 11) support the direct binding of EGCG into the substrate-binding pocket. EGCG forms a hydrogen bond with Lys187 that is part of the catalytic triade of short-chain dehydrogenases, suggesting a direct competition with binding of the substrate and/or cofactor, respectively.

As reported by others [44], many microsomal (drug metabolizing) enzymes (glyceraldehyde-3-phosphate dehydrogenase, CYP1A1, CYP1A2, CYP2B1/2, CYP2E1, CYP3A, catechol-O-methyltransferase and microsomal glutathione transferase 1) are inhibited by EGCG. In these cases, the underlying mechanism of inhibition was suspected to be protein aggregate formation in EGCG-treated microsomes. To exclude protein aggregate formation by EGCG in our studies, 11β-HSD1 was incubated with increasing EGCG concentrations of up to 100 µM for 3 h at 37°C and then analyzed by Western Blot. From our results any crosslinking of purified 11β-HSD1 by EGCG could be ruled out, as no multimeric forms at higher molecular weight regions were observed (Figure 10).

In conclusion, we provide evidence that aqueous extracts of tea *(Camellia sinensis)* are able to inhibit cortisol formation by the enzyme 11β-HSD1. From several abundant constitutents of tea, the major phenolic compound EGCG could be identified as the most potent inhibitor of human 11β-HSD1, with inhibition constants of Ki = 22.68 µM in microsomes and Ki = 18.74 µM for the purified enzyme, respectively. The mechanism of EGCG inhibition is most likely a direct binding to the active site of 11β-HSD1, which is supported by enzyme kinetic studies and a computer aided docking model. Our results decipher the mechanism by which catechins such as EGCG, or green tea in general, have been successfully consumed for thousand of years for general health benefits. These polyphenolic compounds may serve as model structures for the development of novel agents to treat the metabolic syndrome and related diseases.
